# Graphene Oxide Nanosurface Reduces Apoptotic Death of HCT116 Colon Carcinoma Cells Induced by Zirconium Trisulfide Nanoribbons

**DOI:** 10.3390/ijms24032783

**Published:** 2023-02-01

**Authors:** Victor V. Tatarskiy, Olga V. Zakharova, Peter A. Baranchikov, Dmitry S. Muratov, Denis V. Kuznetsov, Alexander A. Gusev

**Affiliations:** 1 Laboratory of Molecular Oncobiology, Institute of Gene Biology RAS, 119334 Moscow, Russia; 2 Institute for Environmental Science and Biotechnology, Derzhavin Tambov State University, 392020 Tambov, Russia; 3 Department of Functional Nanosystems and High-Temperature Materials, National University of Science and Technology «MISIS», 119991 Moscow, Russia; 4 Engineering Center, Plekhanov Russian University of Economics, 117997 Moscow, Russia; 5 Scientific School “Chemistry and Technology of Polymer Materials”, Plekhanov Russian University of Economics, Stremyanny Lane 36, 117997 Moscow, Russia

**Keywords:** graphene oxide, zirconium trisulfide nanoribbons, HCT116 cells, cytotoxicity, toxicity modification

## Abstract

Due to their chemical, mechanical, and optical properties, 2D ultrathin nanomaterials have significant potential in biomedicine. However, the cytotoxicity of such materials, including their mutual increase or decrease, is still not well understood. We studied the effects that graphene oxide (GO) nanolayers (with dimensions 0.1–3 μm and average individual flake thickness less than 1 nm) and ZrS_3_ nanoribbons (length more than 10 μm, width 0.4–3 μm, and thickness 50–120 nm) have on the viability, cell cycle, and cell death of HCT116 colon carcinoma cells. We found that ZrS_3_ exhibited strong cytotoxicity by causing apoptotic cell death, which was in contrast to GO. When adding GO to ZrS_3_, ZrS_3_ was significantly less toxic, which may be because GO inhibits the effects of cytotoxic hydrogen sulfide produced by ZrS_3_. Thus, using zirconium trisulfide nanoribbons as an example, we have demonstrated the ability of graphene oxide to reduce the cytotoxicity of another nanomaterial, which may be of practical importance in biomedicine, including the development of biocompatible nanocoatings for scaffolds, theranostic nanostructures, and others.

## 1. Introduction

Since the discovery of graphene, the number of publications on ultrathin 2D materials in fields such as material sciences, chemistry, nanotechnology, and others has expanded exponentially [1]. This led to the discovery of other 2D nanomaterials [2,3], such as graphene-like materials, di- and trichalcogenides of transition metals [4,5], MXenes [6,7], layered double hydroxide nanosheets [8,9], hexagonal boron nitrides [10], and others [11,12,13].

Among the possible uses of such 2D materials [14,15,16], special attention should be paid to their use in biomedicine [17,18,19,20,21,22]. Due to their chemical, mechanical, and optical properties, 2D nanomaterials are used for drug and gene delivery [23,24,25], tissue engineering [26], bioimaging [27], biosensors [28], antimicrobial and antiviral applications [29,30,31], and cancer therapy [32]. In cancer therapy, 2D materials can be used for drug delivery [33,34], immunotherapy [35], photothermal and photodynamic therapy [36,37], and diagnostics [38,39]. Furthermore, due to their bioactive properties, some materials can also be used for direct treatment of cancer cells [40,41,42]. For example, 2D multilayered nanosheets of Ti_2_NT_x_ MXene showed high toxicity for human malignant melanoma cells (A375) and human breast cancer cells (MCF-7) compared to nontransformed human mammary epithelial cells (MCF-10A) and human immortalized keratinocytes (HaCaT) [43,44]. In another study [45], Ti–Al–C-based MAX phases and Ti_3_C_2_Tx MXenes showed specific toxicity caused by reactive oxygen species in cervical cancer cells (HeLa) in comparison with primary human fibroblasts (MSU_1.1_). 

GO at a concentration of 250 μg/mL reduced the viability of human glioblastoma multiforme U-87 cells, HeLa human cervical carcinoma cells, and CasKi human cervical cells by up to 40% [46]. In human liver carcinoma cells (HepG2), GO and rGO (reduced graphene oxide) had dose-dependent effects [47]. Deduced IC20 and IC50 were ∼8 mg/L and ∼46 mg/L for rGO, respectively, whereas they were ∼10 mg/L and ∼81 mg/L, respectively, for GO. Similar cytotoxicity, DNA damage, and oxidative stress with differential dose dependency were observed for both GO and rGO, but they exhibited distinct mechanisms. Hydrophilic GO showed cellular uptake, NADPH oxidase-dependent ROS formation, and high deregulation of antioxidant/DNA repair/apoptosis-related genes. Conversely, hydrophobic rGO was found to be mostly adsorbed at the cell surface without internalization, ROS generation by physical interaction, poor gene regulation, etc. The cell viability of adenocarcinomic human alveolar basal epithelial cells (A549) treated with 20 μg/mL of GO for 48 or 72 h was slightly reduced, at levels of approximately 88.4% and 85.1%, respectively [48]. Ingenuity pathway analysis showed that SMARCA4, TGF-β1, and TP53 were located at the center of the protein interaction network and were considered key node proteins regulating GO toxicity.

Two-dimensional transition metal dichalcogenides showed low cytotoxicity for human lung carcinoma epithelial cells (A549). Nanosheets of MoS_2_ and WS_2_ did not show activity, even in high concentrations, while WSe_2_ showed dose-dependent effects, lowering viability to 31.8% at a maximum concentration of 400 μg/mL [49].

There are virtually no data on the biological activity of the 2D trichalcogenides of transition metals. In our previous investigation, we found that freshly prepared titanium zirconium trisulfide (ZrS_3_) suspensions in a physiological saline solution with concentrations as high as 1 g/L did not exhibit any toxic effects on *Escherichia coli* bacteria. However, ZrS_3_ suspensions that were stored for 24 h prior to bioluminescence tests were very toxic to the bacteria and inhibited their emission, even at concentrations as low as 0.001 g/L. We explained these observations by the aqueous hydrolysis of ZrS_3_, which resulted in the formation of ZrOx on the surface of the ZrS_3_ particles and the release of toxic H_2_S [50].

Data on the combined activity of GO and metal-containing nanoparticles are inconsistent, with some articles showing that GO can decrease the cytotoxic activity of some nanometal particles [51] and others showing that GO can increase their cytotoxic activity [52]. As a rule, the cytotoxicity of such composites is greater than that of GO alone [53,54]. 

In a number of studies focusing on the cytotoxicity of nanocomposites of GO and metal nanoparticles, the separate components were not analyzed [55,56], so it is not possible to make a definitive conclusion regarding the role of GO in the cytotoxicity of other nanomaterials.

Because the biological activity and anticancer properties of GO used in conjunction with the 2D trichalcogenides of transition metals are unknown at present, we studied the cytotoxicity of GO, ZrS_3_, and a GO/ZrS_3_ composition on colon carcinoma cells (HCT116). We showed that while GO has no effect on cells, ZrS_3_ leads to the induction of programmed cell death via apoptosis; this induction was partially decreased by GO. 

## 2. Results

### 2.1. Nanomaterial Characterizations

**Figure 1:**
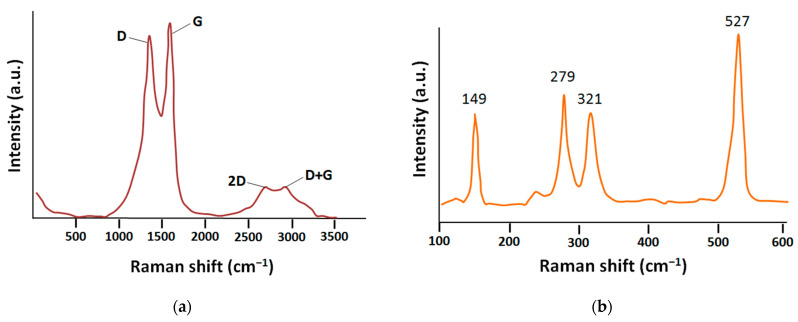
Raman spectra of samples: (**a**) GO; (**b**) ZrS_3_.

As can be seen in Figure 1a, the Raman spectra of the GO samples have four bands: D—1339 cm^−1^, G—1591 cm^−1^, 2D—2680 cm^−1^, and D + G—2980 cm^−1^ vibration modes. The relative intensity of D mode (D/G) is 0.95 and is typical for most GO samples [57,58].

The Raman spectra (Figure 1b) show four modes specific for ZrS_3_: 149, 279, 321, and 527 cm^−1^ [59,60,61], which confirms the presence of these phases in the obtained sample.

**Figure 2:**
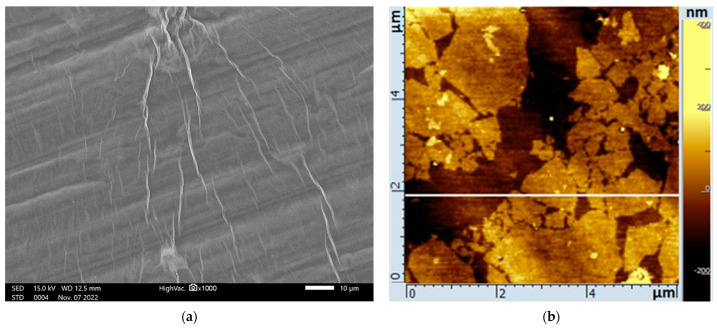

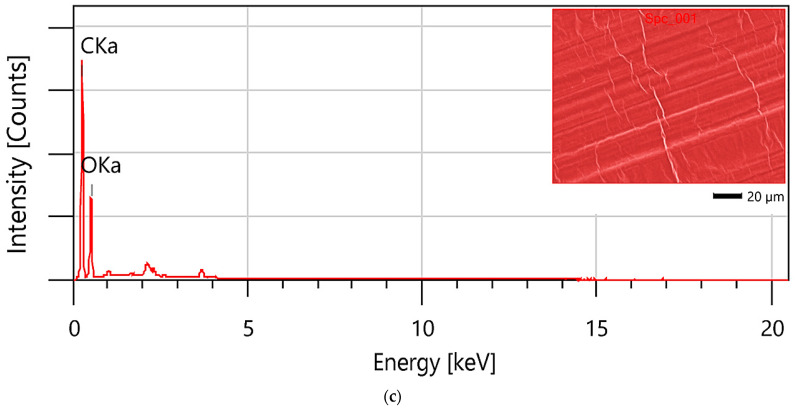
Analyses of GO: (**a**) SEM image; (**b**) AFM image; (**c**) elemental composition (EDX).

Figure 2a shows an SEM microphotograph of a GO film after drying on a silicon wafer, which shows characteristic wrinkles. Atomic force microscopy (AFM) showed that the lateral size of such wafers varied from 0.1 to 3 μm, while their average thickness was less than 1 nm (Figure 2b). The elemental composition of the sample obtained by energy-dispersive X-ray spectroscopy (EDX) is shown in Figure 2c. Apart from carbon and oxygen, no other elements were found, which demonstrated the purity of the nanomaterial.

**Figure 3:**
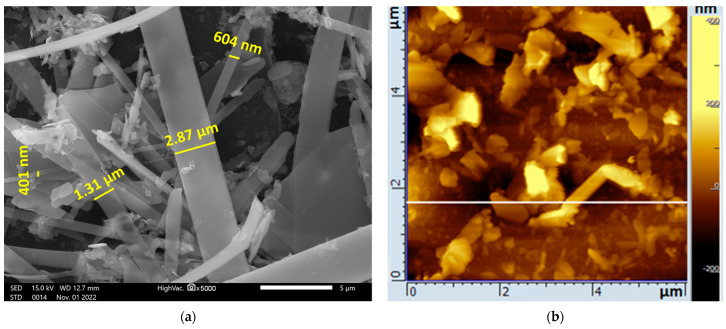

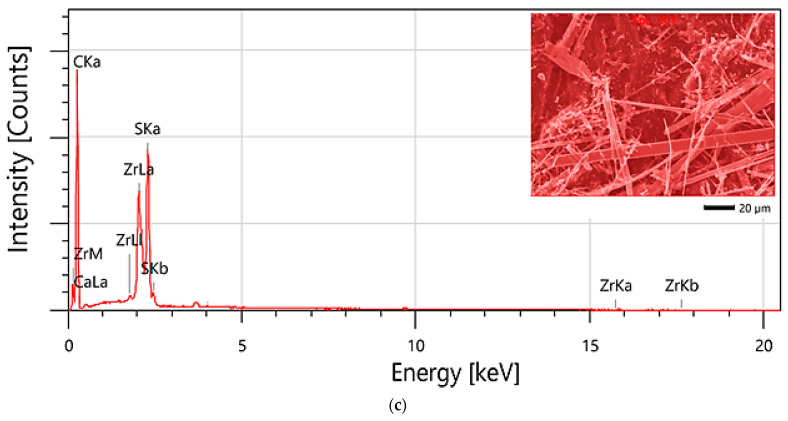
Analyses of ZrS_3_: (**a**) SEM image; (**b**) AFM image; (**c**) elemental composition (EDX).

SEM examination showed that the studied ZrS_3_ nanocrystals after ultrasonic treatment were more than 10 μm in length and 0.4–3 μm in width (Figure 3a). AFM showed that the average thickness of the crystals was 50–120 nm (Figure 3b). The composition of the material included zirconium and sulfur (Figure 3c).

**Figure 4:**
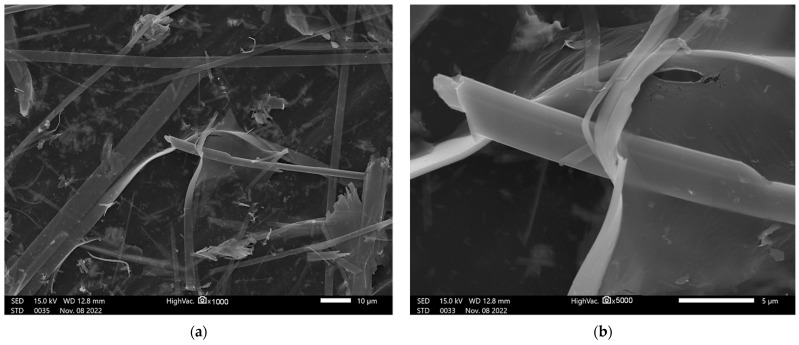

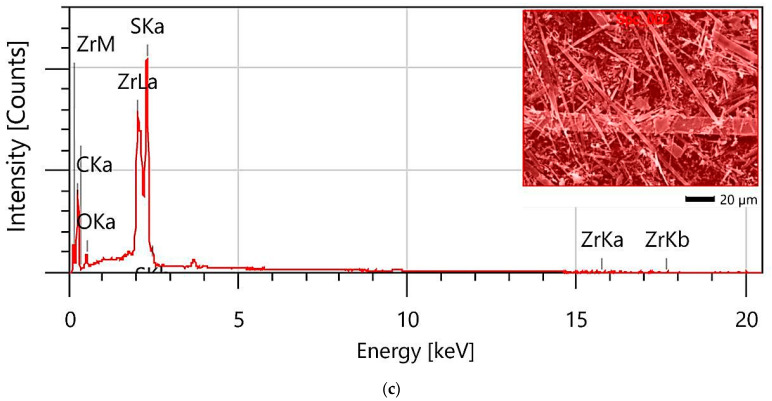
SEM analyses of GO/ZrS_3_: (**a**,**b**) SEM image; (**c**) elemental composition (EDX).

Figure 4a,b show microphotography of films consisting of the ZrS_3_ and GO mix. The microphotographs show GO film covering ZrS_3_ nanocrystals.

The elemental composition of the film is shown in Figure 4c. Energy-dispersive X-ray analysis showed carbon, oxygen, zirconium, and sulfur. 

In summary, the following biological experiments utilized GO nanolayers with dimensions of 0.1–3 μm and an average thickness less than 1 nm together with ZrS_3_ nanocrystals of length more than 10 μm, width 0.4–3 μm, and thickness 50–120 nm. Elemental analysis showed an absence of impurities in the mixture.

### 2.2. Cytotoxicity Assay

#### 2.2.1. Microscopy

**Figure 5:**
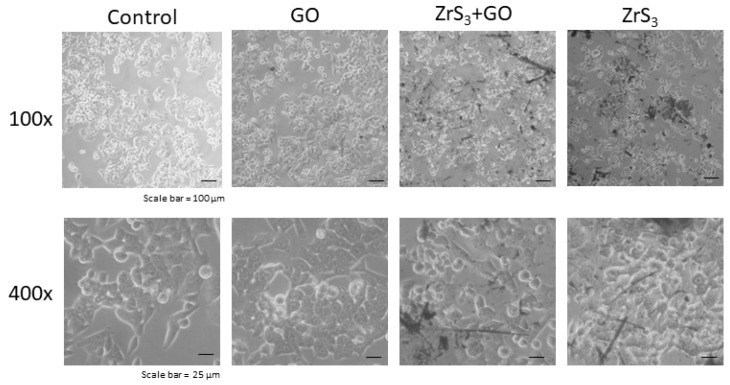
Microphotographs of HCT116 cells growing on nanostructured films.

Figure 5 shows the results of the microscopic analysis of cells cultured on nanomaterial substrates. 

#### 2.2.2. MTT Test

**Figure 6:**
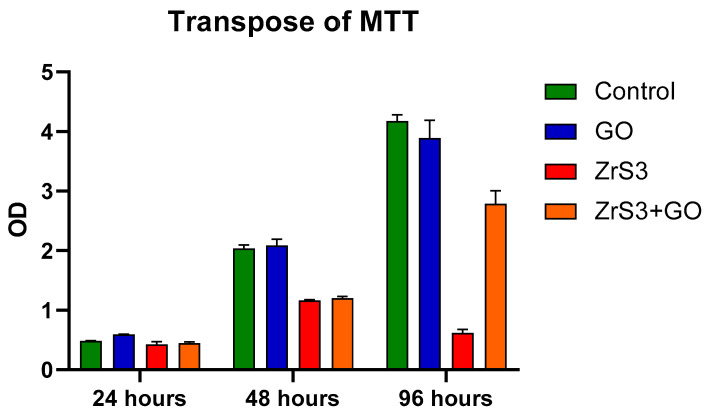
Proliferation of HCT116 cells on nanostructured films (control, GO, ZrS_3_, ZrS_3_+GO).

The influence of nanostructured films on the growth of eukaryotic cells was first assessed in a proliferation assay. Cell growth was monitored using the MTT test, which assesses mitochondrial activity in the cell culture by monitoring the reduction of yellow tetrazolium salt into formazan by mitochondrial dehydrogenases. We used three types of plate well nanocoatings; cells treated with plastic were used as a control. Cells were assayed on days 1, 2, and 4. The largest effect was seen at day 4, with GO having no effect on cell growth, while the other nanostructured films showed a decrease in cell growth. ZrS_3_ by itself completely inhibited cell proliferation, while its mix with GO had less influence on cell growth (Figure 6).

#### 2.2.3. Annexin–PI

**Figure 7:**
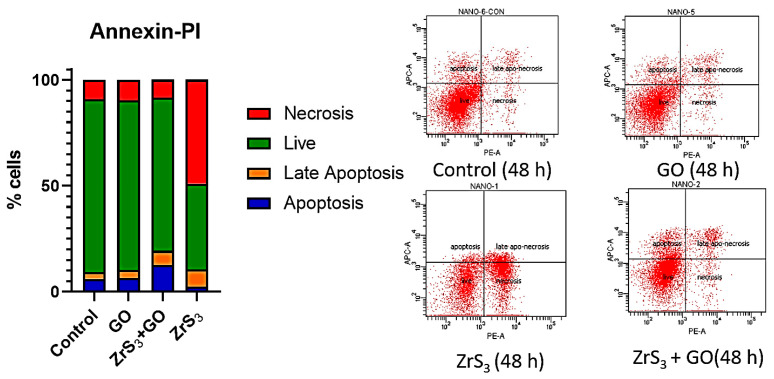
Annexin–PI assay of HCT116 cells on nanostructured films (control, GO, ZrS_3_, ZrS_3_+GO).

Because the MTT test assesses both the inhibition of proliferation and cell death, we cannot discern between the mechanisms by which nanostructured films act on cells. Staining with fluorophore-coupled protein annexin V allows for the detection of cells that have phosphatidylserine in their outer membrane, which is one of the hallmarks of apoptosis. Propidium iodide is a fluorophore that cannot penetrate intact cell membranes but can stain necrotic and late apoptotic (necrotized after apoptosis) cells. We measured staining with annexin V and propidium iodide after 48 h of incubation with HCT116 cells on nanostructured films to measure the induction of apoptosis and necrosis. The ZrS_3_ films, which had the most antiproliferative ability in the MTT test, showed a rapid increase in the necrotic fraction, with no increase in apoptosis and late apoptosis. The mix of ZrS_3_ and GO inhibited the induction of necrosis, switching the mode of cell death to programmed cell death (apoptosis). However, the proportion of early and late apoptotic cells was small (6.5% and 3.7% increases for early and late apoptosis, respectively) (Figure 7).

#### 2.2.4. Cell Cycle

**Figure 8:**
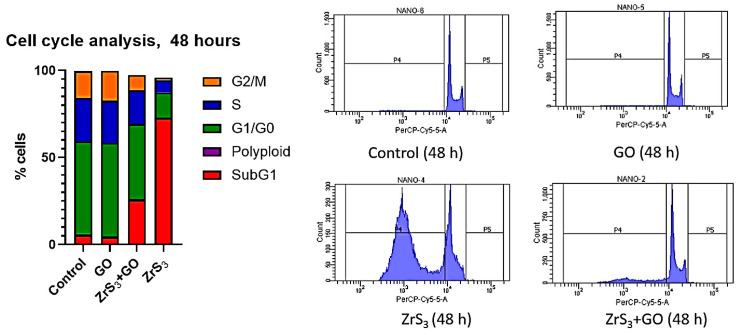
Cell cycle distribution of HCT116 cells on nanostructured films (control, GO, ZrS_3_, ZrS_3_+GO).

Next, we analyzed cell cycle distribution in HCT116 cells to further explore the antiproliferative mechanism of nanostructured films. GO showed the same cell cycle distribution as cells grown on tissue culture treated with plastic, with less than 10% of sub-G1 cells (Figure 8). ZrS_3_-nanostructured film increased the sub-G1 percentage without changing the distribution of the remaining living cells, showing that the mechanisms of the antiproliferative effect of these nanostructured films are most probably direct apoptosis and/or necrosis. The mixing of ZrS_3_ with GO lowered the proportion of the sub-G1 fraction; however, once again, it did not change the cell cycle distribution of the remaining cells. This indicated that nanostructured films act through the direct induction of cell death and do not affect the proliferation of HCT116 cells or induce an interruption of the cell cycle.

## 3. Discussion

We studied the effects that GO nanolayers (of dimensions 0.1–3 μm and thickness of less than 1 nm) and ZrS_3_ nanocrystals (length greater than 10 μm, width 0.4–3 μm, and thickness 5–120 nm) have on the viability, cell cycle, and cell death of HCT116 colon carcinoma cells.

We found that graphene oxide had virtually no cytotoxicity for this cell line. This is consistent with numerous studies that have demonstrated the biocompatibility of graphene-based materials [62,63,64]. For example, it has been shown that graphene films grown by CVD do not inhibit the proliferation of human mesenchymal stem cells or accelerate their differentiation into bone cells [65]. Lasocka et al. found that graphene film did not exhibit cytotoxicity against L929 mouse fibroblasts and increased cell adhesion during 24 h of cultivation [66]. In addition, GO can protect cells from the internalization of toxic hydrophobic molecules, nanoparticles, and nucleic acids, such as siRNA and plasmid DNA, by interacting with cell surface lipid bilayers, without noticeably reducing cell viability [67].

At the same time, zirconium trisulfide nanoribbons had a strong cytotoxic effect on HCT116 cells due to apoptotic cell death. We associated this result with the aqueous hydrolysis of ZrS_3_, which resulted in the formation of ZrOx on the surface of the ZrS_3_ particles and the release of H_2_S [50]. Hydrogen sulfide, an important signaling molecule involved in the regulation of oxidative stress reactions [68], has cytotoxicity associated with the production of ROS [69]. 

Our most interesting conclusion is that the combined use of graphene oxide and zirconium trisulfide was significantly less cytotoxic than zirconium trisulfide alone. Since we associate the toxicity of zirconium trisulfide nanoribbons with the emission of hydrogen sulfide, it can be assumed that graphene oxide reduces this emission by forming a film that is impermeable to H_2_S molecules on the surface of the nanoribbons. It is known that an intact graphene sheet is an effective barrier to gas molecules [70,71]. Figure 4b clearly shows that graphene oxide forms a kind of coating on zirconium trisulfide ribbons, which supports our hypothesis.

Another possible explanation for the molecular mechanism of toxicity modification is the sorption of H_2_S molecules on the surface of graphene oxide nanosheets, which would reduce the concentration of hydrogen sulfide in the medium. This hypothesis is supported by data in the literature. Graphene oxide is a good material for the sorption, storage, and separation of gases [72], including hydrogen sulfide, which has a relatively high bond strength, to the carbon nanostructure [73]. It is also possible that both discussed mechanisms occur simultaneously, or molecular interactions that we have not described are also involved. 

## 4. Conclusions

The exact mechanisms underlying the cytotoxic effects of the studied nanomaterials require further investigation. In any case, using zirconium trisulfide nanoribbons as an example, we have shown that graphene oxide can reduce the cytotoxicity of another nanomaterial, which may be of practical importance for biomedicine (e.g., in the development of biocompatible nanocoatings for scaffolds, theranostic nanostructures, etc.).

## 5. Materials and Methods

### 5.1. Nanomaterial Synthesis

In our research, we used a sample of GO prepared by means of chemical exfoliation of graphite flakes using the Hummers method [74,75,76]. To synthesize ZrS_3_, weighed portions of pure zirconium powder and elemental sulfur powder were taken in a stoichiometric ratio in accordance with reactions (1) [77].

Zr + 3S = ZrS_3_, (1)

All materials were synthesized using reagents from Sigma-Aldrich (Burlington, MA, USA).

### 5.2. Nanomaterial Characterization

Raman spectra were measured using a Thermo Scientific DXR Raman microscope with a 532 nm laser at 1 mW power through a 100× objective. 

The morphology and elemental composition of the nanomaterials were determined using a scanning electron microscope (JCM-7000 NeoScope; JEOL Ltd., Tokyo, Japan).

Atomic force microscopy data for GO and ZrS_3_ were obtained using an AIST-NT instrument (USA) in semicontact tapping mode with NT-MDT AFM tips. 

### 5.3. Nanomaterials Substrate for Cell Cultivations

The GO was resuspended at a concentration of 1 g/L. For stable dispersions of ZrS_3_, 0.01 g of nanomaterial was dissolved in 10 mL of 95% ethanol [78]. Flasks were dispersed in an ultrasonic bath (VBS-41H; Vilitek, Moscow, Russia) for 10 min (power: 180 W, volume 4 L).

A volume of 100 μL of nanomaterial suspension (GO and ZrS_3_) was dispensed into 24 μL plates (100 μL of 1:1 mix of GO:ZrS_3_). To obtain the mixture, 5 mL of GO suspension and 5 mL of ZrS_3_ suspension were added alternately to a 10 mL sterile plastic tube and mixed by vigorous shaking. The plate was left until the suspension dried, and it was then sterilized using UV irradiation for 1 h.

### 5.4. Cells

HCT116 colon carcinoma cells were obtained from the American Type Culture Collection. Cell lines were grown in DMEM medium (PanEco, Moscow, Russia) containing 10% fetal bovine serum (HyClone, GE Healthcare Life Sciences, Chicago, IL, USA), 2 mM L-glutamine, 100 U/mL penicillin, and 100 μg/mL streptomycin (PanEco, Moscow, Russia) (full growth medium) at 37 °C in 5% CO_2_.

### 5.5. MTT Test

The HCT116 cells were seeded at a concentration of 10,000 cell/well in control or film-covered wells in 1 mL of full growth medium and left for 24 h to attach. The cells were analyzed after 24, 48, and 96 h of incubation. For analysis, 5 mg/mL of MTT (Sigma, Burlington, MA, USA) solution was added to the medium, and the cells were left for 2 h of additional incubation at 37 °C in 5% CO_2_ for the staining to develop. Next, the medium was removed, and the stained cells were dissolved in 500 μL of DMSO (PanEco, Moscow, Russia). The optical density was measured at a wavelength of 570 nm using a CLARIOstar Plus (BMG LABTECH, Ortenberg, Hessen, Germany).

### 5.6. Annexin–PI

Apoptosis was analyzed using an Alexa Fluor^®^ 488 Annexin V/Dead Cell Apoptosis Kit (Thermo Fisher Scientific, Inc., Waltham, MA, USA) according to the manufacturer’s protocol. Briefly, HCT116 cells were seeded at a concentration of 100,000 cells/well in a 6-well plate (Corning, New York, NY, USA) and allowed to attach for 24 h. After 48 h of incubation, the cells were carefully detached using a Versen EDTA solution (PanEco, Moscow, Russia), so as not to damage the films. The cells were centrifuged for 5 min at 1500 rpm, washed with PBS (phosphate-buffered saline: 150 mM sodium phosphate and 150 mM NaCl, pH = 7.4), and centrifuged again. The cell pellets were then resuspended in 100 μL Annexin V Binding Buffer (10 mM HEPES, 140 mM NaCl, and 2.5 mM CaCl_2_, pH 7.4). Then, annexin V and propidium iodide were added. The cells were stained for 15 min at room temperature in the dark. After incubation, 400 μL of Annexin V Binding Buffer was added and assayed on a BD FACS Canto II (BD, Franklin Lakes, NJ, USA) flow cytometer using the PE and FITC channels.

### 5.7. Cell Cycle

HCT116 cells were seeded at a concentration of 100,000 cells/well in a 6-well plate (Corning, New York, NY, USA) and allowed to attach for 24 h. After 48 h of incubation, the cells were carefully detached using Versen EDTA solution (PanEco, Moscow, Russia), centrifuged, and washed with PBS. After centrifugation, the cells were lysed in PI lysis buffer containing 50 μg/mL propidium iodide (PI), 100 μg/mL RNAse A (Sigma-Aldrich, Burlington, MA, USA), 0.1% sodium citrate, and 0.3% NP-40 for 30 min in the dark. The cell nuclei were analyzed using a BD FACS Canto II (BD, Franklin Lakes, NJ, USA) flow cytometer in the PE channel.

### 5.8. Data Analysis

Statistical data processing was carried out with Microsoft Excel 2010 (descriptive statistics package) using one-way analysis of variance (ANOVA) with the calculation of Fisher’s F-test at a 5% significance level.

## Data Availability

The data that support the findings of this study are available from the authors.
